# Sensitive Dentine
*Though this paper was prepared independent of the discussion at the Convention, I cannot, after looking over the proof sheets of the proceedings, as reported, refrain from protesting against the gross perversion of my remarks on this subject; and also through a mistake of the printer, the attributing to me observations made by another, (Dr. Dwinelle, I believe,) and lastly, what will, no doubt, surprise Dr. Taft, that a part of my language should appear as if coming from him, though on this point our views are decidedly dissimilar.


**Published:** 1857-01

**Authors:** J. H. M'Quillen


### ARTICLE XIX.

### *Sensitive Dentine.** By J. H. M'QUILLEN.

THIS condition of the dentine, to which the term inflammation is applied by many, has been the subject of much speculation on the part of dental writers, and various arguments have been advanced in support of the existence of dentitis.

* Though this paper was prepared independent of the discussion at the Convention, I cannot, after looking over the proof sheets of the proceedings, as reported, refrain from protesting against the gross perversion of my remarks on this subject; and also through a mistake of the printer, the attributing to me observations made by another, (Dr. Dwinelle, I believe,) and lastly, what will, no doubt, surprise Dr. Taft, that a part of my language should appear as if coming from him, though on this point our views are decidedly dissimilar.

With due respect to the opinions of these gentlemen, it does not appear to me that they have demonstrated their position as an irrefutable one. My object, however, is not to controvert their arguments; but, on the contrary, to present a chain of *negative* reasoning, that I am inclined to believe will satisfy those who have made inflammation a subject of investigation and reflection, of the impossibility of its occurring in this tissue.

If a section of dentine is placed in the field of the microscope, innumerable capillary tubes are observed radiating from the superfices of the pulp cavity; connecting these tubuli is an intervening granular structure, the intertubular tissue. After proceeding a short distance from the pulp cavity, without any change in their relations, the tubuli eventually divide, subdivide, and anastomose with each other; this is much more marked where the enamel and cementum is in contact with the tissue, than at any other point. It is stated by the highest authority on this subject,* that the *largest* tubuli, (and these are not found in the crown, but in the fang, near the apex,) are but $\frac{1}{10000}$th of an inch in diameter.

Having thus briefly described the appearances presented in the field of the microscope, we will consider the manner in which the circulation is carried on through the tissue. In the dentine of human teeth, the presence of blood-vessels has not been satisfactorily demonstrated; and when it is remembered that the capillary attraction resident in the tubuli, added to the *vis et tergo* of the heart and arteries, should be sufficient to effect all the results that could be accomplished by their presence, it would seem a work of supererogation to have placed them there. As evidence in support of this view, I would cite the experiment of immersing one end of a minute-glass tube, held in a vertical position, in a fluid of sufficient tenuity, when, against gravitation, it will be found to ascend some distance up the tube. It is owing to this purely physical force, in connection with the influences exerted by light, and that elective or vital affinity for the nutrient fluid that characterizes all organized

*Tomes, p. 39.

bodies—as evinced in the acts of nutrition and secretion—that the circulation in the vegetable economy is effected.

It may, therefore, I think, be safely concluded that the tubuli—opening as they do upon the walls of the pulp cavity—their contiguity to each other—the division, subdivision and anastomosis—the capillary attraction ; and, added to these, the elective or vital affinity exerted by the tissue—affords the most ample facilities, without the intervention of vessels for the circulation of the *liquor sanguinis*, or nutrient portion of the blood. It is impossible, however, that the *red corpuscles*, which contain the *hematine or coloring matter of the blood*, and whose average diameter is $\frac{1}{4500}$th of an inch, can pass into the tubuli, when the diameter of the *largest* is not one-half that size. Is not this fact conclusive proof of the non-existence of vessels ?

Though not demonstrated by the microscope, it can, with some degree of plausibility, be inferred from the sensibility of the tissue, that nervous filaments from the pulp pass into the tubuli, and form a plexus under the enamel, where the tubuli anastomose with eaeh other.

If then, as I think has been fairly demonstrated, blood-vessels are not present in dentine, how can inflammation possibly occur in it ?

Without intending to enter into a detailed consideration of inflammation, I would now direct attention to the fact, that it is characterized by the presence of four phenomena : pain, redness, heat and swelling. The mere presence of any one of those, however, is not sufficient to establish its existence. As, for instance, *swelling* may be due to emphysema, or dropsical effusion, *preternatural heat*, to friction, exercise, or bringing the part in contact with a fire ; *redness* may arise from exercise, or mental emotions, as in the act of blushing ; and *pain* may be caused by a dislocation, as in colic.

As dentine is of so dense and unyielding a structure, as to preclude the possibility of swelling, and if there is an elevation of the temperature, it is so slight as to be inappreciable to us, redness and pain are the only phenomena that remain to be considered.

A reddened condition of the dentine does occasionally present itself; but this cannot be regarded as proving the presence of inflammation in *that* tissue. As before stated, it is only the *liquor sanguinis* or colorless portion of the blood that can pass into the tubuli; the red corpuscles being too large to enter, and unlike the capillary vessels of the conjunctiva, these tubes are incapable of distention. In the course of my practice I have never met with this reddened condition of the dentine without, at the same time, finding the *dental pulp* in a state of *acute inflammation*, resulting from mechanical violence offered to the tooth, or the improper application of escharotics to obtund the sensibility of the dentine. This redness can only be accounted for by supposing that the *red corpuscles* in the vessels of the inflamed pulp *have been ruptured*, and the *hematine* escaping unites with the *liquor sanguinis*, is carried into the tubuli.

This, then, reduces us to but one of the phenomena, *pain;* and as it has already been shown that the mere presence of only one is not sufficient to establish the existence of inflammation, it is fair to presume that the rule should hold good in this tissue as well as in others.

After a careful examination of the nature of this sensibility, and the influences that develop it, I am disposed to believe that, with some exceptions, it is due to physiological rather than pathological conditions. I will offer a case. A patient presents himself, complaining of tenderness in a tooth, which is not *persistent* in its character, and only manifesting itself when the tooth is subjected to variations of temperature, as the contact of cold air, cold or hot fluids. Upon attempting to remove the decay, it is found to be exquisitely sensitive, though the caries is comparatively superficial; the pain induced by the contact of the instrument, however, ceases almost immediately upon the removal of the excavator from the cavity. In the majority of cases, if the operator persists, after the first few cuts have removed the upper lamina of decay, the sensibility becomes very slight, or disappears altogether; but, in some instances, the tenderness continues during the entire excavation, and even when introducing the filling. If the dentine of a perfectly sound tooth in the mouth of such a person should be suddenly exposed by removal of the enamel, through accident or design, the same intolerance to variations of temperature or the contact of an instrument, would manifest itself at once. Now, all this only proves that the part is endowed with that vital characteristic of the animal kingdom, sensibility, and that the peculiarities of organization render some individual more impressible than others.

If any other part of the body should be subjected to the same influences—for instance, immersing the hand in hot or cold water and retaining it there some time, or cutting it with a knife—a sense of pain would be induced, but no one would dream of attributing it to the existence of inflammation. That such influences will develop inflammation in the tissues capable of such condition is without question; the pain, however, that accompanies its establishment is *persistent* in its character, until either resolution is induced or the part dies; and, though external influences may *aggravate* it, they are not demanded to make it manifest. In sensibility of dentine, on the contrary, as long as these influences are not brought to bear upon the tissue, the pain remains quiescent.

The sensation perceived in the dentine is no doubt due to impressions made upon the nervous filiments, ramifying through the tubuli, and the plexus which is found directly under the enamel, offers such an extended surface of nerve substance as to render that the most sensitive point. The disappearance of the sensibility, after this part is removed, has been accounted for by supposing that the nervous current is destroyed by the section of the filaments.

I have stated before that there are some cases in which we may regard the sensibility as due to pathological conditions. Teeth, for instance, which are perfectly devoid of sensibility, when their possessor is in the enjoyment of health, become exceedingly tender when he is afflicted with disease. Under such circumstances, the blood circulating throughout the entire system is very much altered in its characteristics, and that portion of it which passes into the tubuli may make such an impression upon the nervous filaments as to induce an exalted sensibility in the part, but it cannot be regarded as inflammation.

Aside from the fact that inflammation is accompanied by certain phenomena, it also *terminates* either in *resolution*, *suppuration*, *mortification*, et cetera. There is not satisfactory evidence that any of these have ever presented themselves as sequences, resulting from inflammation in this tissue. There was a period when caries of the teeth was regarded as a result of inflammation, but that opinion of later years has been abandoned, and the theory of chemical decomposition adopted in its place.

The treatment pursued in this condition is one little calculated to *arrest* the progress of inflammation; but, on the contrary, eminently qualified to *increase* it, when applied to a part in which it truly exists. Suppose the same course should be carried out in the treatment of inflammation of bone—the tissue to which dentine bears the closest analogy—first cutting out the inflamed part, and then introducing in its place a filling of gold, tin or amalgam. Would this be likely to stop the disease?

Recapitulating them as follows: I would say that the non-existence of blood-vessels in the tissue; the absence of all the phenomena but one, and that requiring the action of external influences to develop it; the insufficient evidence that any of the results of inflammation have ever presented themselves; and the fact that placing a foreign body, such as gold, tin or amalgam, in contact with a tissue in a state of inflammation, would be calculated to increase rather than arrest it, is sufficient evidence, it appears to me, to prove that inflammation cannot and does not occur in this tissue.

With such facts before me, I can but regard this condition of the dentine as proving the existence of sensibility in the part, and that, owing to peculiarities of organization in some, and certain conditions of the system in others, it occasionally becomes unduly exalted.

Though not prepared to entirely ignore the use of escharotics, to obtund this sensibility, I am satisfied from an experience of nearly ten years, that in the use of one, the chloride of zinc, the remedy is almost as bad as the disease; and in the second, arsenic, that there is very great danger, even in the most careful hands, of exciting inflammation in the pulp, and consequent reddening of the dentine; and a third, the nitrate of silver, there is every probability of inducing a permanent discoloration of the tooth. In the majority of cases, if not in all, I believe they can be dispensed with, by following the course suggested by Dr. Neall, "of applying the keen edge of a well-tempered excavator to the margin of the caries, whipping out in a moment or two the major and more sensitive parts of the decay, cutting as much as possible from centre to circumference, and more towards than from you, putting the patient upon his legitimate endurance, and by a prompt and straightforward manner, securing the like from him."—*Dental News Letter.*

